# Engineered Recombinant Single Chain Variable Fragment of Monoclonal Antibody Provides Protection to Chickens Infected with H9N2 Avian Influenza

**DOI:** 10.3390/vaccines8010118

**Published:** 2020-03-03

**Authors:** Deimante Lukosaityte, Jean-Remy Sadeyen, Angita Shrestha, Joshua E. Sealy, Sushant Bhat, Pengxiang Chang, Paul Digard, Munir Iqbal

**Affiliations:** 1 The Pirbright Institute, Pirbright, Woking GU24 0NF, UK; deimante.lukosaityte@pirbright.ac.uk (D.L.); jean-remy.sadeyen@pirbright.ac.uk (J.-R.S.); angita.shrestha@pirbright.ac.uk (A.S.); joshua.sealy@pirbright.ac.uk (J.E.S.); sushant.bhat@pirbright.ac.uk (S.B.); pengxiang.chang@pirbright.ac.uk (P.C.); 2 The Roslin Institute, University of Edinburgh, Easter Bush, Midlothian EH25 9RG, UK; paul.digard@roslin.ed.ac.uk

**Keywords:** single chain variable fragment antibody (scFv), passive immunisation, recombinant antibodies, neutralizing antibodies, influenza virus, chicken protection

## Abstract

Passive immunisation with neutralising antibodies can be a potent therapeutic strategy if used pre- or post-exposure to a variety of pathogens. Herein, we investigated whether recombinant monoclonal antibodies (mAbs) could be used to protect chickens against avian influenza. Avian influenza viruses impose a significant economic burden on the poultry industry and pose a zoonotic infection risk for public health worldwide. Traditional control measures including vaccination do not provide rapid protection from disease, highlighting the need for alternative disease mitigation measures. In this study, previously generated neutralizing anti-H9N2 virus monoclonal antibodies were converted to single-chain variable fragment antibodies (scFvs). These recombinant scFv antibodies were produced in insect cell cultures and the preparations retained neutralization capacity against an H9N2 virus in vitro. To evaluate recombinant scFv antibody efficacy in vivo, chickens were passively immunized with scFvs one day before, and for seven days after virus challenge. Groups receiving scFv treatment showed partial virus load reductions measured by plaque assays and decreased disease manifestation. These results indicate that antibody therapy could reduce clinical disease and shedding of avian influenza virus in infected chicken flocks.

## 1. Introduction

Recent work on broadly neutralising antibodies has suggested that passive immunisation could be used to tackle viral diseases with potentially greater therapeutic effects than those of antivirals [1,2]. It is a particularly attractive strategy due to a rapid onset of protection and ability to work in those that are immunocompromised or carrying maternally derived antibodies [3,4]. Another advantage of immunotherapeutics includes a maintained ability for natural development of an adaptive immune response to the pathogen whilst nevertheless experiencing minimal morbidity levels, thereby reducing susceptibility to subsequent reinfection [5]. Traditionally, antibody-mediated protection is induced by delivery of the whole immunoglobulin molecule that could act via the antigen binding domain (Fab) physically inhibiting various stages of the virus life cycle, or through fragment crystallisable (Fc) region-mediated recruitment of cellular responses [6,7]. Nevertheless, the presence of the Fc region hinders antibody production, increases the likelihood of antibody immunogenicity in heterologous species and for use in chickens, adds further complications due to insufficiently defined avian Fc receptors and their functions [8,9,10]. Antibody engineering allows the generation of smaller, highly specific and less immunogenic molecules such as single chain variable fragment antibodies (scFvs).

Influenza A viruses are classified into different subtypes according to the genetic and antigenic properties of the major virus surface glycoproteins: haemagglutinin (HA) and neuraminidase (NA). To date, 18 HA and 11 NA subtypes of influenza A viruses have been characterised. Among these, H5, H7 and H9 subtype viruses are regarded as the avian influenza viruses (AIV) with the highest propensity to cause morbidity and mortality in Galliformes and Anseriformes [11]. H5 and H7 subtype viruses are known to exist in both high pathogenicity (HP) and low pathogenicity (LP) phenotypes, whereas H9 viruses are classified as a LP phenotype of AIV, with the representative H9N2 subtype found to be globally enzootic in poultry [12]. Concerns over H9N2 viruses include not only significant risks to poultry industry worldwide, but also because of their demonstrated ability to undergo rapid genetic reassortment, either donating or acquiring internal gene segments, giving rise to new viruses with greater disease risk to other avian and mammalian species including humans [13,14,15,16]. Prevention and control of AIV in poultry remains challenging, particularly in regions such as Asia, Middle East and North Africa where disease is endemic [17,18]. Routinely used inactivated virus vaccines do not guarantee either sterile immunity (even after booster doses) or induce rapid immune responses, limiting vaccine usage to preventative rather than emergency vaccination [19,20]. In addition, generation of antigenic variants under the pressure of inefficient vaccine immunity further decreases vaccine effectiveness [21,22,23].

In this study, as a proof of concept, we assessed whether scFvs can be used for therapeutic purposes via intranasal delivery in birds. We exploited previously generated murine monoclonal antibodies that were shown to neutralize H9N2 virus in vitro [24]. Antigen binding domains of murine antibodies were sequenced; variable heavy (V_H_) and variable light (V_L_) chains of the IgG molecules were used for scFv production, whose protective potential was then successfully evaluated in chickens. We propose this model may also be relevant for other avian viral pathogens.

## 2. Materials and Methods

### 2.1. Ethics Statement

All described animal studies and procedures were carried out in strict accordance with European and United Kingdom Home Office regulations and the Animals (Scientific Procedures) Act 1986 Amendment Regulations, 2012. These studies were carried out under the United Kingdom Home Office approved project license number P68D44CF. Additionally, the work had undergone ethical scrutiny before approval by The Pirbright Institute’s Animal Welfare and Ethical Review Board (AWERB) under the request number AR000992.

### 2.2. Viruses and Cells

All H9N2 viruses, including A/chicken/Pakistan/UDL/01/2008 (UDL-1/08), A/chicken/Pakistan/UDL-02/2008, A/chicken/Egypt/D7100/2013/, A/Chinese Hwamei/Vietnam/38/2006 and A/chicken/Hong Kong/G9/1997, were propagated in 10-day-old specific pathogen-free (SPF) embryonated eggs and titrated by plaque assay on Madin–Darby canine kidney (MDCK) cells (ATCC).

MDCK cells were maintained in Dulbecco’s modified Eagle’s medium (DMEM) supplemented with 10% foetal bovine serum (FBS), 0.1% penicillin G and streptomycin at 37 °C, 5% CO_2_. Drosophila Schneider 2 (S2) cells were obtained from Invitrogen and maintained in Schneider’s insect medium supplemented with 10% FBS at 25 °C.

### 2.3. Sequencing of Hybridoma Clones

V_H_ and V_L_ sequences used in this study were derived from mouse hybridomas, generated using splenocytes of mice immunized with recombinant UDL-1/08 HA protein, Absolute Antibody Ltd., UK. Briefly, mRNA was extracted and converted into cDNA. Rapid amplification of cDNA ends (5′RACE) was used to identify variable heavy chain sequences of antibodies when a user-defined homopolymer tail was added to 3′ ends of cDNA; the subsequent PCR product was subjected to Sanger sequencing [25]. To determine variable light domain sequences, a V-region PCR approach was undertaken using forward primers specific to signal peptide or framework region 1 and reverse primers corresponding to antibody constant regions were used for product amplification followed by Sanger sequencing [26].

### 2.4. Production and Purification of Recombinant Antibodies

Recombinant scFv antibodies were produced and purified using a Drosophila Expression System (DES^®^, Life technologies, Paisley, UK). Briefly, V_H_ and V_L_ domains of three different mouse monoclonal antibodies (mAbs) derived from sequenced hybridoma clones—F965-ID2, F965-JF7 and F965-EC12—[24] were linked with a (Gly_4_Ser)_4_ linker. A CD33 signal sequence (*Homo sapiens*; accession number NM_001772.4), was fused to the 5′-end of the scFv ORF, while a four codon long (EPEA) C-tag sequence was added to the 3′-end to allow for affinity purification. The expression cassettes were commercially synthesised (GeneArt, ThermoFisher Scientific, Regensburg, Germany) and cloned into pExpres2.1 expression vector (ExpreS2ion Biotechnologies, Hørsholm, Denmark). The recombinant plasmids were transfected into Drosophila S2 cells using Calcium Phosphate Transfection Kit (Thermo Fisher Scientific, Paisley, Scotland, UK). Antibiotic selection was carried out for four weeks using Zeocin (InvivoGen, Toulouse, France) at a concentration of 750 µg mL^−1^ for the first two weeks and 1500 µg mL^−1^ for the next two weeks prior to single cell clone isolation via limiting dilution. Clones showing highest levels of antibody expression by ELISA were expanded and secreted antibodies were purified via the CaptureSelect™ C-tag Affinity Matrix (ThermoFisher Scientific). The concentration of purified recombinant antibodies was determined by Pierce^TM^ BCA Protein Assay Kit (ThermoFisher Scientific) and the purity was assessed by sodium dodecyl sulphate–polyacrylamide gel electrophoresis (SDS-PAGE).

### 2.5. ELISA

Briefly, Nunc^TM^ MicroWell^TM^ 96-well plates (LifeTechnologies) were precoated with live UDL-1/08, A/chicken/Pakistan/UDL-02/2008, A/chicken/Egypt/D7100/2013/, A/Chinese Hwamei/Vietnam/38/2006 and A/chicken/Hong Kong/G9/1997 viruses starting with 64 HAU which were diluted 2-fold and left at 4 °C overnight. Plates were washed with washing buffer (PBS with 0.1% Tween (LifeTechnologies)) prior to blocking with 5% BSA for 1 hour at room temperature. To test binding of scFvs, each antibody was added at a concentration of 1 µg mL^−1^ followed by 1 h incubation at room temperature. Bound scFvs were probed with CaptureSelect™ Biotin Anti-C-tag Conjugate (LifeTechnologies), which was detected using HRP-conjugated streptavidin (LifeTechnologies). Four washes with washing buffer were performed between each step. All incubations were done for 1 hour at room temperature. HRP was detected using 3,3′,5,5′-tetramethylbenzidine (TMB) substrate (BD Biosciences, Wokingham, UK). Reactions were stopped by 2 M H_2_SO_4_ and plates were read at dual wavelength at 450 nm with 630 nm reference in an ELx808 Absorbance Microplate Reader (BioTek, Swindon, UK). The variable heavy chain of the heavy chain only antibody wR1a-12 that was specific to the H7 HA (gift by Hufton et al., National Institute for Biological Standards and Control, UK) and non-binding UDL-1/08 was used as a negative control.

### 2.6. Virus Microneutralisation (MNT) Assay

MDCK cells were pre-seeded into 96-well plates to reach 90–95% confluency. On the day of experiment, antibodies were twofold serially diluted in 60 µL volume (starting at 1 mg/mL) in triplicates and mixed with an equal volume of UDL-1/08 virus containing 150 TCID_50_ (50% tissue culture infective dose). Cells were washed with PBS and inoculated with 50 µL antibody–virus mixture for 1 h at 37 °C. Cells only, virus only and positive H9N2 serum collected from birds challenged with UDL-1/08 at day 14 post-infection (animal experiment request number AR000562) controls were included into each plate. After the incubation, cells were washed once again with PBS and serum free DMEM containing 2 µg mL^−1^ TPCK trypsin was added, cells were left at 37 °C for 72 h. After 3 days medium was removed and cells stained in crystal violet solution for 30 min.

### 2.7. Haemagglutination Inhibition (HI) Assay

World Health Organisation guidelines were followed for HI assays [27]. Briefly, 2-fold dilutions of scFvs were prepared in V-bottom 96-well plates to which 4 HA units of virus was added. The plate was incubated for 1 h at 37 °C. Subsequently, chicken red blood cells (1%vol/vol) were added and results were read after 45 minutes incubation at room temperature. The first dilution of the antibody to result in clear pelleting of the red blood cells was recorded as the end-point dilution.

### 2.8. Animals and AIV Challenge

Mixed sex, SPF eggs of Rhode Island Red chickens resourced from the National Avian Research Facility (Roslin Institute, UK) were hatched at The Pirbright Institute and challenged at 3 weeks of age. Birds were separated into five groups (*n* = 20/group): (group-1) non-treated and challenged with UDL-1/08; (group-2) scFv JF7 (200 µg/dose) treated and challenged with UDL-1/08; (group-3) scFv EC12 (300 µg/dose) treated and challenged with UDL-1/08. Group-4 had 6 birds that were scFv EC12-treated and non-challenged and group-5 had 10 non-treated and non-challenged birds. In each group receiving virus, birds were subdivided into two subgroups: a directly inoculated group (*n* = 10) that were inoculated with 5 × 10^5^ plaque forming units (PFU) of virus by the intranasal route and a contacts group (*n* = 10) left as naïve for virus transmission analysis. Each directly inoculated and contact bird was treated with scFvs by intranasal route 24 h before the challenge as a prophylaxis with the treatment being continued daily until 7 days postinoculation. Four birds per group were sacrificed at day 4 postinoculation and remaining birds were humanely killed at day 14 postinoculation when the experiment was terminated. Chickens were monitored daily for clinical signs and weight changes throughout the experiment.

### 2.9. Sample Collection and Tissue Homogenisation

Swab samples from buccal and cloacal cavities were collected daily from each bird until day 7 postinoculation with the last sampling performed on day 10 post virus inoculation. Sterile polyester tipped swabs were transferred into the virus transport media (WHO, 2006) [28], vortexed and centrifuged for 10 min at 4500 rpm to clarify the medium, samples were stored at −80 °C until further analysis.

On day 4 postinoculation, 4 birds per group were humanely killed to collect nasal turbinates, trachea, lungs, spleen and cecal tonsils that were stored in 10% neutral buffered formalin, RNA later or snap frozen. Twenty milligrams of tissue was used for homogenisation in 1 mL of PBS by TissueLyser LT (Qiagen, Hilden, North Rhine-Westphalia, Germany). Homogenate was clarified by centrifugation and serially titrated by plaque assay. Clarified tissue homogenate was also used for RNA extraction.

### 2.10. Plaque Assay

To determine virus titre from allantoic fluid, swab samples or animal tissues, pre-seeded 12-well plates with MDCK cells were inoculated with 10-fold serially diluted samples and left for 1 h at 37 °C. Cells were washed with PBS and overlaid with flu overlay media (1x MEM, 0.21% BSA, 1 mM L-glutamate, 0.15% sodium bicarbonate, 10 mM Hepes, 0.1% penicillin G/streptomycin) containing 0.6% purified agar (Oxoid) and 2 µg mL^−1^ TPCK trypsin. Cells were left at 37 °C for 72 h. After 3 days medium was removed and cells were stained in crystal violet solution for 30 min.

### 2.11. qRT-PCR of Viral M Gene and Cytokine mRNAs

RNA from swab and tissues samples was extracted using an RNeasy kit (Qiagen) according to the manufacturer’s instructions. Quantification of the viral M gene and specific cytokine mRNAs was performed using single-step real-time reverse transcription PCR with Superscript III Platinum One-Step qRT-PCR kit (LifeTechnologies) with the cycling conditions as per manufacturer’s protocol in a 7500 fast real-time PCR machine (Applied Biosystems, Applied Biosystems Limited, Warrington, UK). For influenza virus, M gene-specific primers and Taqman probes were used as described by Speckman et al., 2002, [29] (M F – AGATGAGTCTTCTAACCGAGGTCG; M R – TGCAAAAACATCTTCAAGTCTCTG; M probe – TCAGGCCCCCTCAAAGCCGA). A T7 RNA polymerase-transcribed RNA standard for the M gene was run alongside for standard curve generation. For cytokine quantification, the primers and probes were used as described by Kapczynski et al. [30] (IL6 F- GCTCGCCGGCTTCGA; IL6 R - GGTAGG TCTGAAAGGCGAACAG; IL-6 probe - AGGAGAAATGCCTGACGAAGCTCTCCA; IFNγ F – GTGAAGAAGGTGAAAGATATCATGGA; IFNγ R – GCTTTGCGCTGGATTCTCA; IFNγ probe – TGGCCAAGCTCCCGATGAACGA; IL-4 F – AACATGCGTCAGCTCCTGAAT; IL-4 R – TCTGCTAGGAACTTCTCCATTGAA; IL-4 probe AGCAGCACCTCC CTCAAGGCACC). For the cytokine analysis, 28S rRNA house-keeping gene was included per sample as an internal standard (28S F – GGCGAAGCCAGAGGAAACT; 28S R – GACGACCGATTTGCACGTC; 28S probe – AGGACCGCTACGGACCTCCACCA). An arbitrary value of 40 was chosen for data transformation as elsewhere [31].

### 2.12. Virus Sequencing

Viral RNA extracted from swab samples was subjected to cDNA synthesis and PCR amplification using Superscript III one-step RT-PCR System with Platinum^TM^ *Taq* DNA polymerase (Invitrogen) in accordance with manufacturer’s protocol and primers as following F – AGCAAAAGCAGGGG; RV – AGTAGAAACAAGGGTGTTTT based on the study of Hoffmann et al. [32]. For amplification of HA gene specific fragment 2 x PfuUltra II Fusion HS DNA Polymerase master mix (Agilent) was used with 0.2 μM (F -TGTAAAACGACGGCCAGTCAACAAGCAATGCAGATAAAATCTGC; R - CAGGAAACAGCTATGACCGCTAATTATATACAAATGTTGCACCTGC) of each primer and cycling conditions (as follows; 95 °C–2 min, 35 cycles of 95 °C–20 sec, 55^+0.2^ °C–20 sec, 72 °C–2 min and final extension 72 °C–3 min. PCR products were purified using 1% agarose gel (Molecular Biology Grade Agarose (Eurogentec) electrophoresis followed by a QIAquick Gel purification Kit (Qiagen) to ensure a single product and sequenced via Sanger sequencing commercially (Source Bioscience, Cambridge, UK). All sequences were analysed using MEGA6: Molecular Evolutionary Genetics Analysis Version 6.0.

### 2.13. Statistical Analysis

All analyses were performed using GraphPad Prism 8 (GraphPad software GraphPad Software, Inc., San Diego, CA, USA). Statistical tests included one-way ordinary ANOVA and Kruskal–Wallis tests. *p*-values < 0.05 were considered significant. Nonsignificant (Ns) = *p* > 0.05; * = *p* ≤ 0.05; ** = *p* ≤ 0.01; *** = *p* ≤ 0.001; **** = *p* ≤ 0.0001. GraphPad Prism 8 was also used for curve fitting following nonlinear regression model.

## 3. Results

### 3.1. scFvs can Bind to and Neutralize H9N2 Virus In Vitro

**Figure 1:**
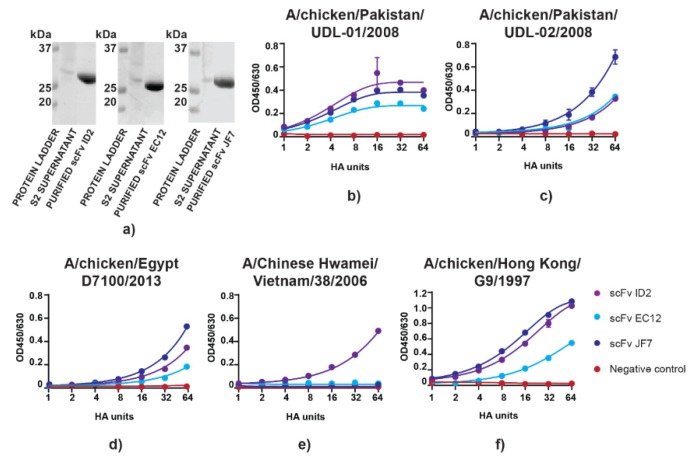
scFv purification and binding to H9N2 viruses. (**a**) SDS-PAGE analysis of purified scFv antibodies. Molecular weight markers 37, 25 and 20 kDa; (**b**) UDL-1/08; (**c**) A/chicken/Pakistan/UDL-02/2008; (**d**) A/chicken/Egypt/D7100/2013/; (**e**) A/Chinese Hwamei/Vietnam/38/2006; (**f**) A/chicken/Hong Kong/G9/1997 virus at serial dilutions starting with 64 HAU. To check antibody affinity, scFvs were purified and used at a constant concentration of 1 µg mL^−1^. scFvs were then probed with anti C-tag antibody conjugated to biotin which was detected using HRP-conjugated streptavidin. The variable heavy chain of the heavy chain only antibody wR1a-12 specific to H7 HA non-binding UDL-1/08 was used as a negative control. Binding was measured in technical duplicate and the average OD450/630 nM was plotted with error bar representing +/-SD, curve fitting was performed following nonlinear regression model.

Recombinant monoclonal antibodies produced in S2 cells were tested for their ability to bind and neutralize H9N2 virus. Plasmids containing scFv expression cassettes of ID2, EC12, and JF7 mAbs specific to the UDL-1/08 H9N2 HA head domain were transfected into *Drosophila melanogaster* S2 cells and the resulting supernatants containing secreted antibodies were used for C-tag affinity purification [24]. Purifications yielded on average 10 mg protein per litre of supernatant which, when assessed for purity by SDS-PAGE, revealed single polypeptide bands that migrated at the expected ~30 kDa size for scFvs (Figure 1a). We then compared virus antigen binding activity between the different recombinant purified scFvs. ELISAs were performed using serially diluted live H9N2 viruses of different strains probed with a constant concentration of purified scFvs. Out of five viruses tested, two (A/chicken/Pakistan/UDL-02/2008 and A/chicken/Egypt/D7100/2013) shared >95% amino acid identity with UDL-01/08 (96% and 97%, respectively); another two viruses (A/Chinese Hwamei/Vietnam/38/2006 and A/chicken/Hong Kong/G9/1997) had 92% amino acid similarity to UDL-01/08, whereas A/turkey/Wisconsin/1/1966 was the most diverged with 87% amino acid identity. Importantly, A/Chinese Hwamei/Vietnam/38/2006 showed highest divergence at the residues accounting for antigenic positions predicted to be involved in antibody binding [24]. In general, scFv ID2 and scFv JF7 showed higher affinity to the antigen as compared to scFv EC12 which gave a lower plateau for UDL-1/08, A/chicken/Egypt/D7100/2013, and A/chicken/Hong Kong/G9/1997 (Figure 1a,c,f). On the other hand, there was no difference between scFv ID2 and scFv EC12 binding to A/chicken/Pakistan/UDL-02/2008, whereas scFv JF7 showed greater affinity to this antigen (Figure 1c). Only scFv ID2 retained binding activity to A/Chinese Hwamei/Vietnam/38/2006 (Figure 1e). All scFvs retained their binding activity to viruses that had >90% amino acid similarity to UDL-1/08 virus, whereas no binding was detected for the highly diverged H9N2 A/turkey/Wisconsin/1/1966 virus (data not shown).

Next, functional activity of scFvs was assessed by MNT and HI assays using UDL-1/08 as the model H9N2 virus. Herein, scFv JF7 showed weaker activity in the HI assay than the ID2 and EC12 scFvs (Table 1).

However, scFv JF7 exhibited superior activity in the more biologically relevant MNT, where it performed markedly better than the other two constructs. When combinations of the scFv antibodies were tested, none resulted in increased MNT titres (data not shown). Overall, all scFvs retained reactivity against UDL-01/08 virus, although assay-specific variations in titres were seen.

### 3.2. Antibody Therapy Results in Lower Morbidity Levels in Chickens

**Figure 2:**
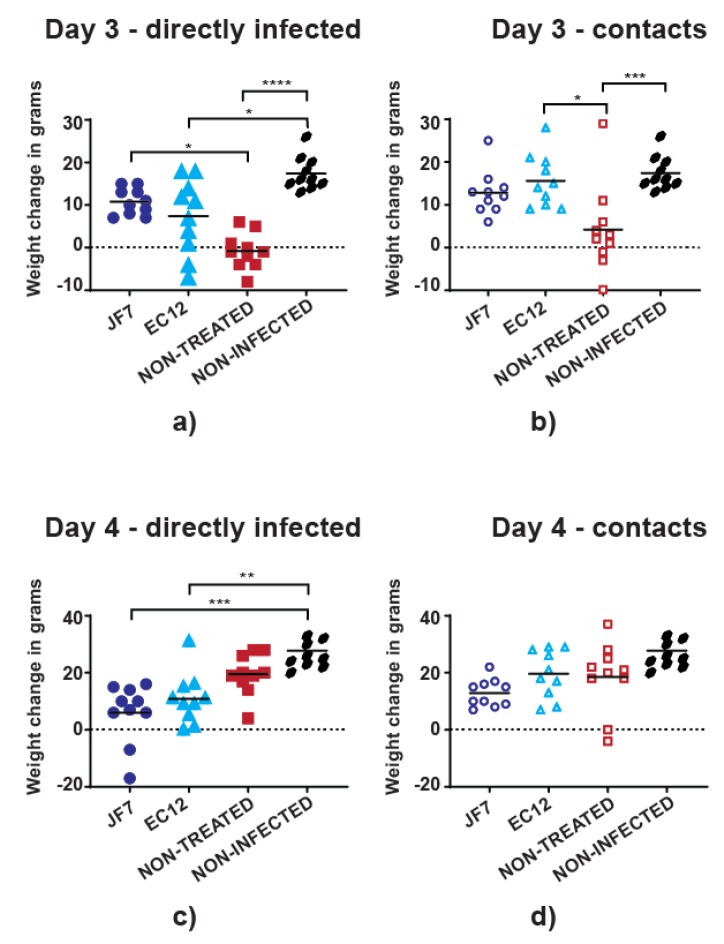
scFv JF7 and scFv EC12 efficacy in chickens. Birds received scFv EC12 (300 µg/dose) or scFv JF7 (200 µg/dose) by intranasal route 24 hours before challenge with 5 × 105 PFU of UDL-1/08 followed by daily treatment till day 7 post virus inoculation. Body weight changes recorded in grams on days (**a,b**) 3 and (**c,d**) 4 post virus inoculation in (a,c) directly infected and (b,d) contact birds; non-infected and non-treated birds were used as a control with each dot representing an individual bird. Levels of significance were based on *p*-values from Kruskal–Wallis test (* = *p* ≤ 0.05; ** = *p* ≤ 0.01; *** = *p* ≤ 0.001; **** = *p* ≤ 0.0001).

We next examined if scFv antibody therapy could protect chickens against disease. Either of scFvs EC12 (300 µg/dose) or JF7 (200 µg/dose) was used as a prophylaxis 24 hours before challenge with H9N2 UDL-1/08 (5 × 10^5^ PFU), followed by continued daily treatment till 7 days post virus inoculation. The different treatment doses of the two scFvs were selected due to their differential activity in vitro so that averaged dose per group would not dramatically exceed 1mg/kg during the initial treatment (representative averaged values for scFv JF7 – 0.666 mg/kg and scFv EC12 – 1.008 mg/kg). Birds were subdivided into 5 groups, 3 of which received virus and either of the treatments or no treatment at all. An additional 2 groups contained non-challenged and non-treated birds or non-challenged but treated with scFv EC12 chickens serving as controls. Virus infection did not cause visible clinical symptoms in treated or control groups throughout the experiment, but there were differences in weight gain patterns at the peak of infection (as defined by virus shedding; see later) on days 3 and 4. Among the directly infected non-treated birds, 70% (7/10) lost or did not gain weight at day 3 post-challenge, and a similar trend was seen in 30% (3/10) of the birds that received scFv EC12 (Figure 2a).

In contrast, all birds that received scFv JF7 had gained weight at day 3 post virus inoculation. This resulted in a significant difference between non-treated challenged birds and the ones that were infected and received scFv JF7. Nevertheless, 2 birds from the directly infected group that received scFv JF7 lost 7g and 17g of weight resulting in 2.59% and 5.23% total body weight loss, respectively, at day 4 post-challenge opposed to no weight loss noted in scFv EC12-treated or non-treated groups, where up to 11% body weight gain was observed (Figure 2c). Furthermore, birds that were contacts to the directly inoculated chickens and were treated with scFv EC12 or scFv JF7 showed ever-growing weight gain whilst 30% (3/10) and 10% (1/10) of non-treated contact birds had reduced weight on days 3 and 4 post-challenge, respectively (Figure 2b,d). Dynamics of the overall weight changes in percentage also reflected halt in weight gain of non-treated chickens infected either directly or by contact at day 3 post virus inoculation as compared to treated birds (Figure S1a,b). However, non-treated birds were quickly recovering at day 4 post-challenge. Neither directly infected nor contact birds that were treated with scFvs JF7 or EC12 had such an apparent hinder in weight gain and were continuing to increase in body mass at a steady rate. Thus, treatment with scFvs conferred partial protection from disease morbidity in both directly infected and contact birds.

### 3.3. scFv Treatment Reduces Peak Virus Shedding

**Figure 3:**
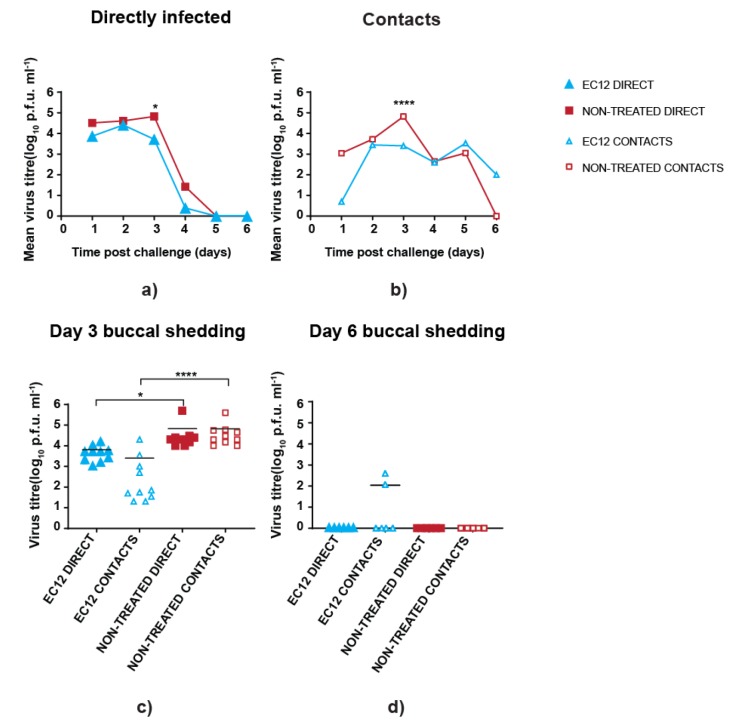
Virus shedding from buccal cavity in scFv EC12-treated and non-treated birds. Chickens were infected with 5x105 PFU of UDL-1/08 and daily administered with 300µg scFv EC12 by intranasal route staring at day −1 and continuing until day 7 post-challenge. Viral titres were determined by plaque forming assay. (**a**) Mean infectious virus titres from buccal swabs of directly infected birds. (**b**) Mean infectious virus titres from buccal swabs of contact birds. (**c**) Buccal shedding titres from directly infected and contact birds at day 3 post-challenge with each dot representing individual bird. (**d**) Buccal shedding titres from directly infected and contact birds at day 5 post-challenge with each dot representing individual bird. Levels of significance were based on *p*-values from Kruskal–Wallis test (* = *p* ≤ 0.05; **** = *p* ≤ 0.0001).

To determine if scFv EC12 could limit virus replication in birds, we first analysed virus loads in swab samples from the buccal cavity. Marginally lower titres of infectious virus particles in pre-treated directly infected birds were noted on day 1 post-challenge (Figure 3a).

Similarly, on average, a reduced virus load was observed in treated contact birds, although the differences were non-significant when compared with the non-treated counterpart on the first two days after infection (Figure 3b). However, virus titres from day 3 samples showed that treated birds in both directly infected and contact groups had, on average, at least ten-fold reduced titres in comparison to non-treated birds (Figure 3c). Following on, titres shed by all directly infected birds (treated or not) dropped sharply at day 4 post virus inoculation and were undetectable on days 5 and 6. In contrast, contact infected birds shed at moderate level (~1100–3300 PFU/mL) for 5 days. Untreated birds cleared virus by day 6, whereas scFv EC12-treated birds continued to shed low levels of virus (~100 PFU/mL) (Figure 3b). This persistence of virus on day 6 evident in EC12-treated contact birds was driven by a few birds and not the whole group (Figure 3d).

**Figure 4:**
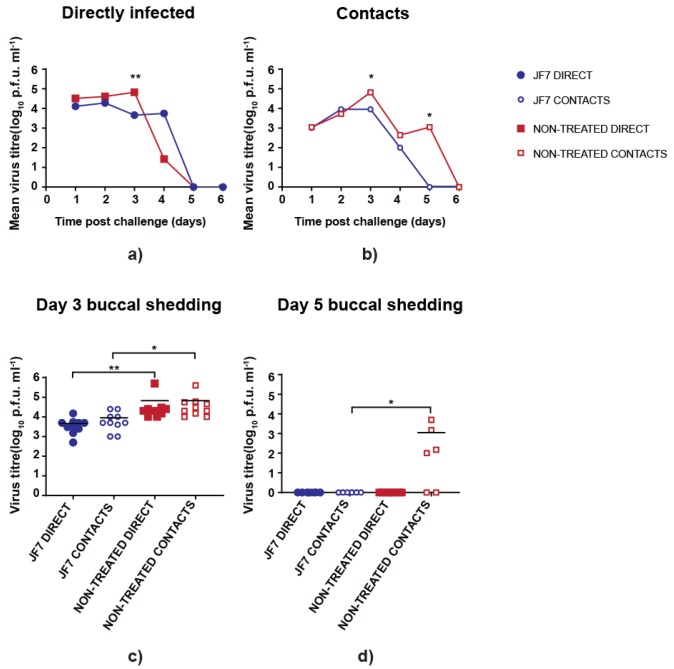
Virus shedding from buccal cavity in scFv JF7 treated and non-treated birds. Chickens were infected with 5 × 10^5^ PFU of UDL-1/08 and daily administered with 200µg scFv JF7 by intranasal route staring at day −1 and continued until day 7 post-challenge. Viral titres were determined by plaque forming assay. (**a**) Mean infectious virus titres from buccal swabs of directly infected birds; (**b**) Mean infectious virus titres from buccal swabs of contact birds; (**c**) Buccal shedding titres from directly infected and contact birds at day 3 post-challenge with each dot representing individual bird; (**d**) Buccal shedding titres from directly infected and contact birds at day 5 post-challenge with each dot representing individual bird. Levels of significance were based on *p*-values from Kruskal–Wallis test (* = *p* ≤ 0.05; ** = *p* ≤ 0.01). Note that non-treated bird data are replotted from Figure 3.

To further assess the therapeutic efficacy of scFv JF7, we similarly measured virus shedding from the buccal cavity of infected birds. As already seen, titres from untreated directly infected birds peaked at day 3 post-challenge and then declined sharply, whereas average titres from scFv JF7 treated birds remained relatively constant but lower until day 4 post (Figure 4a). In both treated and non-treated directly inoculated groups virus was cleared at the same point, with no infectious particles found at day 5 postinoculation (Figure 4a).

Differences between treated and untreated groups were observed in the contact birds, which showed both lower and a reduced period of virus shedding in the treated birds (Figure 4b). Significantly higher virus titres were present in the non-treated group infected either directly or by contact on day 3 postinoculation than in treated birds, whereas non-treated contacts were still shedding virus on day 5 postinoculation (Figure 4c,d). Thus, overall, intranasal delivery of scFv JF7 can suppress viral replication providing partial protection for birds.

In addition, for both treated groups and non-treated controls only sporadic shedding of virus was detected from the cloacal cavity, with the titres being consistently low amongst all groups and with no statistically significant differences recorded (data not shown). To assess tissue tropism of the virus, nasal turbinates, trachea, lungs, spleen and cecal tonsil tissues were collected at day 4 postinoculation, with infectious virus recovered only from respiratory tract, nasal turbinates and trachea; again with no differences seen between groups (data not shown).

### 3.4. Non-Treated Birds Have Higher Inflammatory Responses in the Spleen

**Figure 5:**
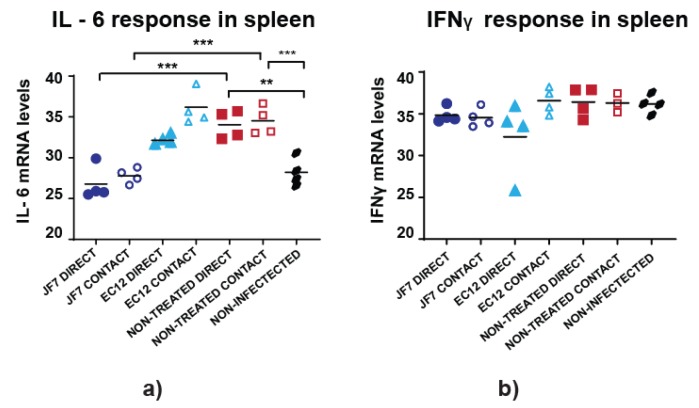
Cytokine mRNA expression in chickens treated with scFvs. RNA was extracted from bird tissues and qRT-PCR performed for the indicated transcripts as well as 28S rRNA as a reference gene. Values are represented as 40-ΔCT values after normalisation to 28S rRNA. (**a**) IL-6 levels in spleens; (**b**) IFNγ levels in spleens. Cytokine expression for each bird is represented in a single dot. A one-way ordinary ANOVA test was run for statistical analysis. *p*-values of *P* < 0.05 were considered significant (** = *p* ≤ 0.01; *** = *p* ≤ 0.001).

To further evaluate the effects of scFv treatment on virus infection, levels of the proinflammatory gene transcripts encoding IL-6 and IFNγ were analysed at day 4 post-challenge in nasal turbinates, trachea, lungs, spleen and cecal tonsil tissues, likely representing major sites of infection or immune response. Data analysis indicated minimal variation in cytokine patterns with no detectable response over the background of uninfected group birds in nasal turbinate, trachea, lung and cecal tonsil tissues (data not shown). The only differences found when comparing 40-ΔCT values were in IL-6 responses in spleens, considered to be peripheral lymphoid organs. Non-treated birds infected either directly or via contact had significantly higher IL-6 levels compared to non-infected group or challenged birds receiving scFv JF7 treatment (Figure 5a).

However, there were no differences found between scFv EC12-treated and non-treated birds regardless on the route of infection. Such pattern was also absent in IFNγ responses (Figure 5b). Overall, elevated IL-6 activity found in the spleens of non-treated H9N2 virus infected birds suggests a widespread inflammatory state within this group, whereas scFv JF7 treatment prevented this inflammatory response in directly infected and contact birds.

### 3.5. scFv Treatment Leads to Virus Escapes

**Figure 6:**
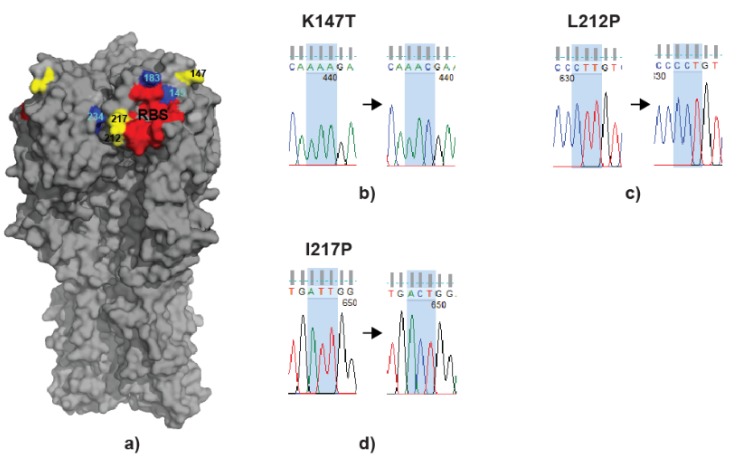
Locations of mutant residues and sequencing traces. (**a**) HA head is in grey, receptor binding site (RBS) in red with highlighted residues P92, G128, T129, S130, S131, A132, W143, N173, V180, L184, Y185, N214, G215, L216, G218 and R219. Mutations recorded in this study are in yellow at positions 147, 212 and 217. Mutations found previously with monoclonal antibodies derived from hybridoma clones F965-ID2, F965-JF7 and F965-EC12 are in blue. Numbering is based on mature H9 protein (PDB ID 1JSD) (**b**) K147T resulting from one nucleotide substitution; (**c**) L212P resulting from one nucleotide substitution; (**d**) I217P resulting from one nucleotide substitution.

To follow on, virus recovered from the swab samples at day 4 postinoculation of birds confirmed to carry the highest virus load from each group was sequenced for HA gene. In short, this included 5 samples (2 direct and 3 contacts) from the group treated with JF7, 6 samples (2 direct and 4 contacts) from the EC12-treated group and 4 (2 direct and 2 contacts) from the non-treated group. In addition, the virus used for inoculation was also sequenced and served as a control. Sequences recovered from inoculum as well as from untreated birds’ swabs were found to match the NCBI database sequence (accession number CY038466). On the other hand, samples from treated birds confirmed the same three mutations L212P, I217P and K147T appearing on day 4 postinoculation in 1 (directly infected) out of 5 samples chosen for the JF7 treated group and 2 (1 direct and 1 contact) out of 6 samples in the EC12-treated group. Two of the mutations identified in this study, L212P and I217P, were located close to the HA receptor binding site and had been previously seen in immune escape variants (together with other amino acid substitutions at positions 145, 183 and 234) when UDL-1/08 virus was cultured in vitro in medium containing the respective mABs derived from hybridomas [24], whereas K147T appeared only in vivo (Figure 6).

These results suggest that treatment with scFvs can reduce viral load but virus escape mutants are likely to emerge.

## 4. Discussion

A range of antibodies targeting all three influenza A virus surface proteins (HA, NA and matrix protein) have been demonstrated to be effective when used for treatment in animal models including mice, ferrets or primates, with some antibodies entering efficacy evaluation in swine and even humans [1,3,5,31,33,34,35]. Nevertheless, HA remains the main target protein for antibodies displaying therapeutic efficacy due to the antigen availability on the virus particle surface. Antibodies that target HA can be categorised into two major groups: Broadly neutralizing antibodies, including MEDI8852, CR6261 or F16 bind, to the conserved HA stem, often resulting in interference with virion fusion, whereas antibodies such as those described in this paper target the HA head and are thought to obstruct receptor binding [36,37,38,39,40]. The major drawback of broadly neutralizing antibodies is caused by limited accessibility of the target epitopes, which can compromise antibody function, thus presumably necessitating higher doses for administration. In addition, such antibodies often rely on effector functions displayed by the Fc part of the immunoglobulin which might not be compatible in different host species [31,41]. Although HA-targeting antibodies can accelerate virus evolution and generation of escape mutants, it is also thought that the combination of several mAbs recognising different epitopes could reduce the level of antigenic changes and thus improve treatment potency [42]. In addition, recent work by Bangaru et al. describes a previously undetected antibody class recognizing a conserved epitope within the HA head, possibly indicating another readily available site for virus life cycle obstruction whose mutation would penalise viral fitness [43].

Within this study, the therapeutic efficacy of scFvs targeting the H9 HA head was explored in poultry.

We had previously generated a panel of mouse monoclonal antibodies [24], which, in this study, were engineered to scFvs and tested for their neutralizing activity against H9N2 representative UDL-1/08 virus. In agreement with other studies investigating monoclonal antibody efficacy [3,44], we also found that scFvs retained protective and therapeutic efficacy in vitro and in vivo. Although binding avidity of scFv JF7 to the antigen might has been impacted by lost antibody bivalence or other structural changes it maintained superior neutralizing activity. With the anticipation that antibody therapy could be used for poultry, we selected chickens as an animal model because they are an economically and epidemiologically important host for AIV infection and thus requires protection from disease [45,46,47]. We found that treatment with scFvs resulted in reduced viral load when compared to non-treated birds during the critical stages of infection. Although differences were marginal during the initial days of infection, directly infected birds that received scFv EC12 showed an earlier infection peak with a lower amount of virus shedding through buccal cavity in comparison to the control group. In addition, virus shedding period was reduced in scFv JF7 treated birds that were infected through contact. However, note that mutant viruses, likely to represent antigenic escape variants, were recovered in some of the birds treated with scFvs. Similar outcomes describing escape mutants arising at day 5 post-infection with lower virus titres maintained at the beginning of the challenge in monoclonal antibody treated animal models were noted by Itoh et al. [3]. It remains to be determined if combination of the observed mutations L212P, I217T and K147T in the challenge virus can affect viral fitness in both naïve and antibody treated birds. Nevertheless, often such changes induced by mAbs can be predicted beforehand using standard in vitro techniques permitting selection of the antibody combination least likely to generate escape mutants. Therefore, these preliminary results in our study support the assumption that H9 HA targeting scFvs can be a valuable tool for the disease control in poultry flocks.

We hypothesised that intranasally administered H9 HA-specific scFvs could prevent or limit virus infection, leading to reduced disease symptoms in chickens. Intranasal antibody administration has previously been shown to provide superior protection in mice when compared to systemic delivery, as well as to require lower doses to achieve protective effects [5,48,49]. An alternative delivery method could be via food pallets as some evidence exists on virus replication in gastrointestinal (GI) tract of poultry; however, in most of the cases, LPAI H9N2 poses higher tropism for respiratory tract in chickens and the effect of low pH in GI on antibody stability remains elusive [50,51,52]. On the other hand, antibody delivery platform via aerosol would significantly reduce labour costs and would also ensure more effective distribution of the molecules into the target organs including lungs [53]. In addition, such approach is already in use for mass vaccination of poultry flocks. Although scFv half-life in birds was not investigated in this study, we anticipate it could be a limiting step that decreases treatment efficacy. Therefore, for future work, it would be beneficial to attempt vectored immunoprophylaxis, as this has previously proven to be efficacious [54,55]. Although in our hands, intranasal antibody administration to the chickens resulted in altered viral shedding kinetics, likely due to presence of antibody in nasal passages where the virus naturally replicates, it remains to be determined which application method would be the most useful for implementation on farmed animals. In addition, note that the timing and length of the treatment might be crucial for the success of the antibody therapeutic efficacy, with many reports emphasising the importance of the timing of the first dose [5,56].

Analysis of the cytokine pattern changes in influenza infected animals receiving antibody therapeutics is not well described in the literature. In this study we wanted to ensure scFvs would not interfere with cellular response involvement and to determine any underlaying signs of infection that could not be assessed by virus titration. We saw elevated IL-6 levels in spleens of non-treated birds which we concluded to be signs of more widespread inflammation. This agreed with the general consensus suggesting IL-6 upregulation in internal organs, including the spleen in H9N2 infected birds, as well as with a study in primates where cytokine levels were assessed in serum samples [3,57]. In agreement with Itoh et al., we were not able to detect IL-4 production at day 4 post virus inoculation, although, in our study, qRT-PCR was used (data not shown), this is likely to correspond with previously described IL-4 downregulation in H9N2 infected chickens [3,58].

## 5. Conclusions

In conclusion, antibody therapy is a promising approach to tackle avian influenza virus and potentially other viral pathogens. Due to inadequate virus control by vaccination in many underdeveloped countries, farmers are willing to use any available measures. Although regulations prohibit antiviral use for farmed animals, there is increasing evidence of such malpractice taking place, leading to generation of resistant viruses [59,60,61]. Such a burden could be possibly reduced by introduction and availability of immunotherapeutics which could also play role in containment of new outbreaks. Herein, we have shown that recombinant scFvs produced in insect cell culture system can be an alternative control measure with no need of antibody grafting to match species of interest. This study suggests that antibody treatment can reduce morbidity symptoms including weight loss and viral load in infected birds.

## Figures and Tables

**Table 1:** scFv antibody activity in virus microneutralisation and haemagglutination assays. To assess scFv functional activity both assays were run in technical and biological duplicates. Average data is shown with ± SD where applicable.

scFv	MNT Titre/mg	HI Titre/mg
**ID2**	59 (±61)	30,720
**EC12**	384 (±222)	2560
**JF7**	4096	160

## Supplementary Materials

The following are available online at https://www.mdpi.com/2076-393X/8/1/118/s1, Figure S1: Percentage (%) body mass changes of challenged chickens. Birds received scFv EC12 (300 µg/dose) or scFv JF7 (200 µg/dose) by intranasal route 24 h before challenge with 5 × 105 PFU of UDL-1/08 followed by daily treatment till day 7 post virus inoculation. Weight of chickens was measured on days 2, 3 and 4 post-challenge and changes were calculated as % of initial weight recorded on day 2 post virus inoculation. (**a**) Directly infected bird weight gain and (**b**) contact bird weight gain.
