# Supplementary material for: Engineered Recombinant Single Chain Variable Fragment of Monoclonal Antibody Provides Protection to Chickens Infected with H9N2 Avian Influenza
Source: Vaccines (Basel). 2020 Mar 3;8(1):118. doi: 10.3390/vaccines8010118 (PMC7157677; doi:10.3390/vaccines8010118)
Supplement: Supplementary file 1 [file vaccines-08-00118-s001.pdf]

Supplementary

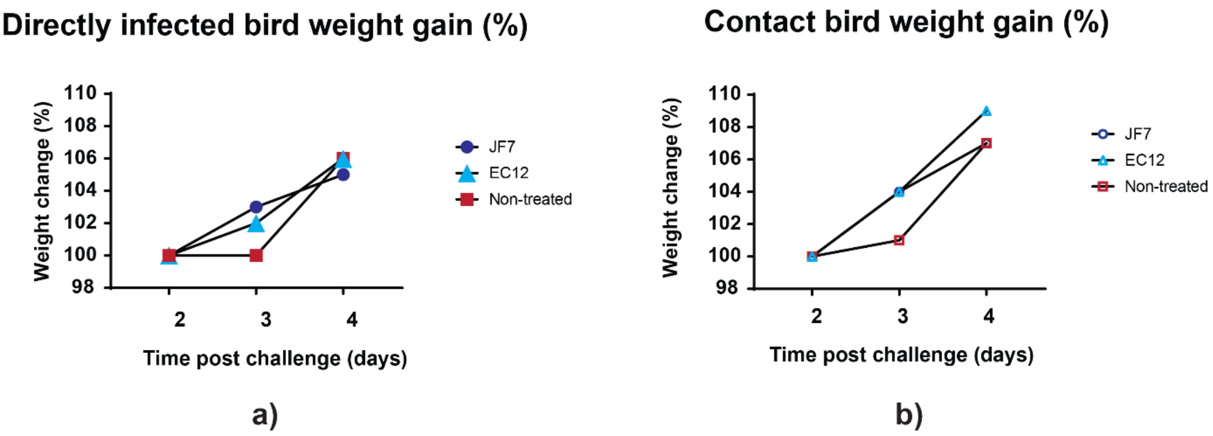

Figure S1. Percentage (%) body mass changes of challenged chickens. Birds received scFv EC12 (300µg/dose) or scFv JF7 (200µg/dose) by intranasal route 24 hours before challenge with 5×10^5 PFU of UDL-1/08 followed by daily treatment till day 7 post virus inoculation. Weight of chickens was measured on days 2, 3 and 4 post challenge and changes were calculated as % of initial weight recorded on day 2 post virus inoculation. (**a**) directly infected bird weight gain and (**b**) contact bird weight gain.
